# Assessment of Antibiotic Sensitivity in Biofilms Using GelMA Hydrogel Microspheres

**DOI:** 10.3390/gels12010085

**Published:** 2026-01-18

**Authors:** Junchi Zhu, Wenqi Chen, Zhenzhi Shi, Yiming Liu, Lulu Shi, Jiafei Xi

**Affiliations:** 1 The Research Institute of Advanced Technologies, Ningbo University, Ningbo 315211, China; 2311100407@nbu.edu.cn (J.Z.); 2411100322@nbu.edu.cn (W.C.);; 2 Beijing Institute of Radiation Medicine, Beijing 100850, China

**Keywords:** biofilm hydrogel beads, antibiotic susceptibility assays, biofilm phenotype, antibiotic resistance, hydrogel

## Abstract

Conventional antibiotic susceptibility testing (AST) primarily assesses planktonic bacteria. However, the three-dimensional architecture and barrier properties of biofilms mean that the minimum inhibitory concentration (MIC) for planktonic cells is typically far lower than the antibiotic exposure required for biofilm eradication. In this study, gelatin methacryloyl (GelMA) microspheres were used to create a three-dimensional biofilm microenvironment for the quantitative evaluation of biofilm tolerance. *Escherichia coli* K-12 MG1655 was immersed in GelMA microspheres and subjected to a series of antibiotic concentration gradients. Bacterial viability was inferred from time-dependent changes in microsphere diameter. The results demonstrated substantial tolerance of the resulting biofilms to ampicillin, ciprofloxacin, and ceftriaxone, with biofilm antibiotic tolerance values exceeding 200 μg/mL, 10–50 μg/mL, and 20–50 μg/mL, respectively. Relative to planktonic MICs, these tolerance levels are elevated by one to two orders of magnitude and surpass the standard clinical breakpoint thresholds. This methodology includes a high-throughput platform, involving only several hundred microspheres and achieving completion within 24 h, thereby offering a useful platform for investigating biofilm resistance mechanisms and guiding antibiotic treatment strategies.

## 1. Introduction

Antibiotic resistance has become a serious threat to global human health. It is estimated that at least 700,000 deaths occur worldwide each year due to drug-resistant infections [1]. Among the factors contributing to enhanced resistance, the formation of microbial biofilms plays a major role. Microbial biofilms confer resilience against environmental stresses, protecting bacteria from fluctuations in pH, osmolarity, and mechanical forces [2,3]. The attachment of bacteria to surfaces triggers rapid changes in the expression of genes associated with biofilm maturation and exopolysaccharide (EPS) production. These genetic alterations facilitate massive bacterial colonization on both biotic and abiotic surfaces, leading to the formation of a protective barrier [4,5,6]. Conventional clinical antimicrobial susceptibility testing (AST) is typically performed on planktonic (free-living) bacteria [7]. However, treatment strategies based solely on planktonic AST results show a success rate of only 8–30% [8]. Given the urgent need to combat antimicrobial resistance, it is crucial to develop novel testing methods that incorporate native biofilm environments into antimicrobial efficacy assessments.

The current gold standard methods for AST in clinical and laboratory settings are based on planktonic culture systems, completely overlooking the unique growth characteristics of biofilms. Although current biofilm models have advanced to include various systems such as 96-well microplates [9], modified Robbins devices [10], Calgary biofilm devices [11], rotating disk reactors [12] and CDC biofilm reactors [13], these models still exhibit significant limitations. In vivo, biofilms typically form within complex microenvironments, particularly in high-viscosity conditions (e.g., synovial fluid), where bacteria can establish dispersed and heterogeneous three-dimensional (3D) aggregate structures [14]. The sparse and non-uniform nature of such in vivo biofilms significantly reduces the predictive efficacy of conventional planar culture-based AST methods. Consequently, there is an urgent need to develop integrated biofilm models that can accurately recapitulate in vivo 3D microenvironments to establish a new biofilm-oriented AST standard with high clinical relevance.

Recent advances in hydrogel technology have revolutionized biofilm research through engineered matrices like agarose, chitosan and sodium alginate [15,16]. Of particular significance, gelatin methacryloyl (GelMA) combines tunable physicochemical properties with biomimetic extracellular matrix (ECM) architecture [17,18], enabling fabrication of 3D microenvironments that recapitulate critical biofilm characteristics: matrix porosity [19], oxygen gradients [20], and antibiotic diffusion barriers [21]. This system not only encapsulates bacteria with high fidelity but also serves as a versatile platform for studying biofilm control strategies under clinically relevant conditions. With its unique ability to mimic biofilm characteristics and broad biomedical compatibility, GelMA has emerged as a groundbreaking tool for fundamental and translational microbiology research [22,23].

In this work, we established a GelMA microsphere-based system for antibiotic susceptibility assessment, in which the diameter variation in *E. coli*-encapsulating microspheres is tracked upon antibiotic treatment. The testing approach incorporates key biofilm characteristics, including their 3D structure and diffusion-limiting matrix, thereby yielding physiologically informative results with potential clinical utility.

## 2. Results and Discussion

### 2.1. Biofilm Formation on GelMA Microspheres

**Figure 1:**
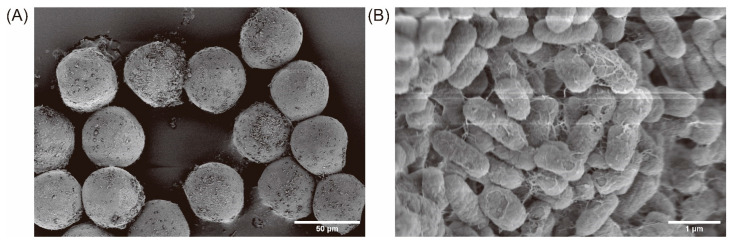
Scanning electron microscope (SEM) images of *E. coli* MG1655 on GelMA microsphere surfaces. Scale bars: (**A**) 50 μm and (**B**) 1 μm.

To assess the attachment and biofilm formation of *E. coli* MG1655 on GelMA microspheres, the bacteria-incubated microspheres were processed by critical point drying and imaged using scanning electron microscopy (SEM). Observations showed that the GelMA microspheres exhibited a well-defined spherical morphology with a diameter of 50–100 μm and possessed a porous surface architecture. The microspheres were well dispersed and maintained structural integrity after bacterial incubation. Bacterial attachment was evident across multiple regions of the microsphere surface, indicating favorable interactions between the bacteria and the GelMA matrix (Figure 1A). At higher magnification (Figure 1B), mature *E. coli* MG1655 biofilms were clearly observed covering the microsphere surface. Individual rod-shaped bacterial cells were embedded within a dense extracellular polymeric substance (EPS) network, with scattered EPS residues preserved following ethanol dehydration, consistent with previously reported biofilm morphologies [24]. Collectively, the data demonstrate that GelMA microspheres support bacterial adhesion, growth, and biofilm formation by *E. coli* MG1655, establishing a robust platform for downstream biofilm-oriented antibiotic susceptibility analyses.

### 2.2. Size Difference Between Empty Beads and Bacteria-Loaded Beads

**Figure 2:**
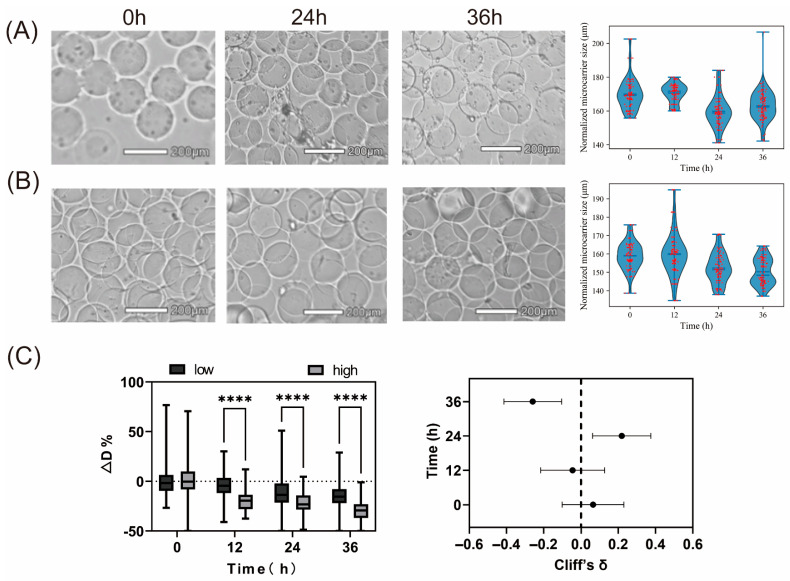
(**A**) Microscopic images and microsphere size distribution of *E. coli*–loaded microspheres and (**B**) control (blank) microspheres. On the **left**, bright-field microscopy images of microspheres at different time points are presented, with their size distribution on the **right**. (**C**) **Left**: Box plot of ΔD%, comparing *E. coli*–loaded and blank microspheres at different time points (0, 12, 24, 36 h). **Right**: Forest plot showing the effect size (Cliff’s δ) with 95% bootstrap confidence intervals (CI) indicated by horizontal lines. Each group consisted of approximately *n* = 100 microspheres analyzed at each time point. (**** *p* < 0.0001).

To evaluate the impact of *E. coli* MG1655 adhesion on microspheres, changes in microsphere diameter during incubation were quantitatively analyzed. As incubation progressed, bacteria continuously proliferated on the microsphere surfaces, accompanied by a gradual decrease in microsphere diameter, with a more pronounced reduction observed at 36 h (Figure 2A). Specifically, the average diameter of microspheres in the bacteria-inoculated group decreased from 158.94 ± 7.67 μm at 0 h to 152.33 ± 8.22 μm at 24 h and further to 150.82 ± 7.40 μm at 36 h, corresponding to an overall reduction of approximately 5.1%. By comparison, microspheres in the control group exhibited only minor diameter fluctuations during incubation (Figure 2B), with the average diameter remaining within a narrow range (169.98 ± 10.15 μm at 0 h and 164.58 ± 6.15 μm at 36 h). Consistent with these observations, boxplot analysis revealed a substantial diameter reduction in the presence of bacteria, as indicated by δ ≈ −0.26 and q ≈ 0.005 at 36 h (Figure 2C). One plausible mechanism involves the secretion of proteolytic enzymes, such as metalloproteases and serine proteases, together with acidic metabolic byproducts, including lactic and acetic acids, by *Escherichia coli* during proliferation [25]. Proteases—including matrix metalloproteinases (MMPs)—are capable of degrading collagen, while acidic microenvironments accelerate GelMA gel hydrolysis; collectively, these processes contribute to a reduction in microsphere size [26]. Notably, microsphere shrinkage can still occur even when intact but metabolically inactive bacterial cells remain embedded within the gel matrix. Therefore, changes in microsphere diameter represent an integrated response that reflects biofilm viability, mechanical reorganization, and degradation of the gel matrix. Furthermore, as *E. coli* forms biofilms on GelMA microspheres, it releases extracellular polymeric substances, including polysaccharides and proteins, potentially modifying surface hydrophilicity and causing microsphere structural failure. Therefore, variations in microsphere diameter upon bacterial colonization serve as a sensitive, visual, and quantifiable indicator, offering a reliable metric for subsequent intervention studies such as antibiotic treatment

### 2.3. Impact of Antibiotic Exposure on E. coli Growth and Hydrogel Microsphere Size

**Figure 3:**
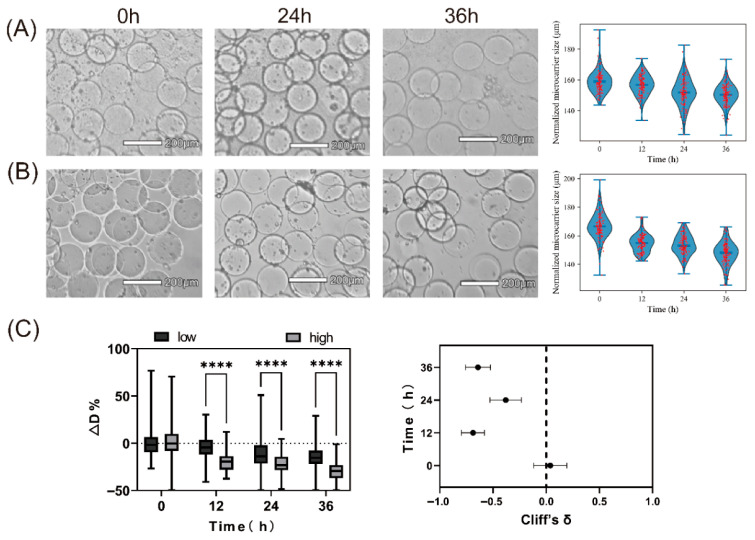
Diameter changes in *E. coli*–laden GelMA microspheres under various antibiotic concentrations. (**A**) Low-dose condition (5 μg/mL CEF) and (**B**) high-dose condition (50 μg/mL CEF) with bright-field images (**left**) and corresponding microsphere size distributions (**right**, violin plots) at various time points. Scale bar: 200 μm. (**C**) ΔD% box plot (**left**) and effect size forest plot (**right**) comparing *E. coli*–loaded microspheres and blank microspheres at various incubation times. (**** *p* < 0.0001).

These results indicate that once *E. coli* adheres to GelMA microspheres, it forms biofilms that consequently decrease the microsphere diameter. Therefore, to further examine the changes in bacteria-laden microspheres upon antibiotic exposure, we incubated them in ceftriaxone (CEF) solutions at 50 μg/mL (high dose) and 5 μg/mL (low dose), and measured diameter changes over time. The results showed that under low antibiotic concentration (5 μg/mL), the microsphere diameter gradually decreased over time, reaching approximately a 10% reduction at 36 h (Figure 3A). When exposed to a higher antibiotic concentration (50 μg/mL), the decrease in the diameter of the microspheres was more significant (Figure 3B). Previous studies have shown that antibiotic exposure often results in growth arrest, structural reorganization, and partial loss of biofilm biomass rather than complete biofilm eradication [27,28]. Such antibiotic-induced structural remodeling may cause measurable compaction of the biofilm–gel composite, manifested as a reduction in microsphere diameter [29,30]. Meantime, CEF may inhibit cell wall synthesis in subpopulations of *Escherichia coli*, leading to increased protease release and accelerated hydrogel degradation. Moreover, exposure to high antibiotic concentrations can induce bacterial SOS stress responses, which may stimulate excessive secretion of degradative enzymes to acquire essential nutrients, thereby enhancing degradation of the amino acid components of GelMA.

GelMA provides a hydrated and compliant three-dimensional microenvironment that supports bacterial adhesion and surface-associated growth, conditions known to promote biofilm formation and enhance antibiotic tolerance compared with planktonic cultures [31]. Meanwhile, a GelMA concentration of 3% (*w*/*v*) provides an appropriate balance between mechanical stability, porosity, and permeability, making it suitable for biofilm formation. At this concentration, GelMA hydrogels exhibit mesh size and stiffness comparable to those reported for native biofilm matrices, while retaining sufficient compliance to allow bacterial adhesion, extracellular matrix development, and antibiotic diffusion. GelMA is generally regarded as chemically inert toward most antibiotics and does not participate in covalent interactions. In our system, biofilms predominantly form on the surface of the microspheres, while antibiotics are applied from the surrounding solution. As a result, antibiotic exposure occurs mainly at the biofilm–liquid interface, and diffusion through the hydrogel bulk is not the primary transport pathway. In addition, GelMA microspheres do not alter the intrinsic antibiotic susceptibility of bacteria; rather, they promote surface-associated biofilm formation, leading to an apparent increase in antibiotic concentrations required for growth inhibition, consistent with biofilm-associated tolerance [32].

### 2.4. Assessing the Tolerance of E. coli Biofilms to Various Antibiotics via GelMA Microspheres Platforms

When planktonic bacteria develop into biofilms, their antibiotic resistance increases dramatically. Our findings indicate that GelMA microspheres colonized by *E. coli* develop surface biofilms and undergo diameter changes in response to antibiotic exposure. Accordingly, this assay was used to evaluate the tolerance of biofilm-associated *E. coli* to three representative antibiotics: ampicillin (AMP), ciprofloxacin (CIP), and ceftriaxone (CEF). Previous literature suggests that biofilm-associated bacteria can demonstrate antibiotic tolerance increases of 10 to 1000 times [33].

**Figure 4:**
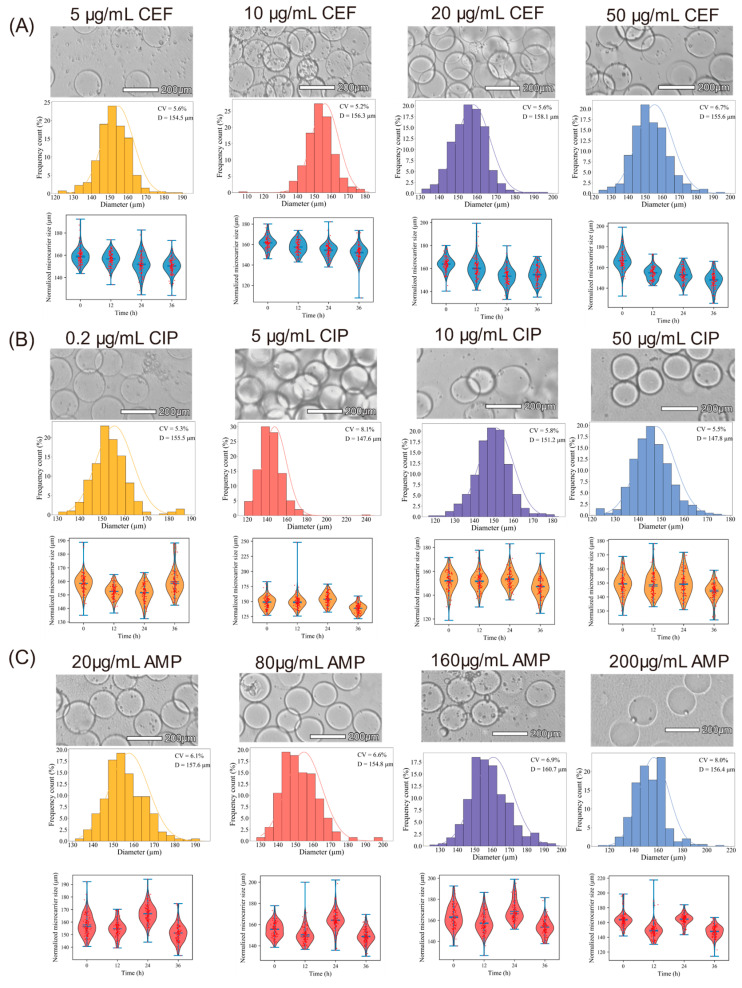
Particle size distribution variations in *E. coli* MG1655-loaded GelMA microspheres at different time points during antibiotic exposure. Microspheres treated with (**A**) ceftriaxone (CEF), (**B**) ciprofloxacin (CIP), and (**C**) ampicillin (AMP) for 0, 12, 24, and 36 h, with images (**top**), size distribution histograms (**middle**), and violin plots (**bottom**) illustrating the changes. Each microsphere is represented by a point; vertical bars denote the observed range. Data were collected using high-throughput bright-field imaging and automated segmentation; only microspheres that met the geometric inclusion criteria were analyzed, and only those with sphericity values (S < 0.05) were considered (Sphericity < 0.05). D is the average maximum diameter for each condition; CV refers to the coefficient of variation in microsphere size. Scale bar: 200 µm.

As shown in Figure 4A, CEF exposure produced a substantial left shift in diameter distribution along with an increased CV, reflecting significant microsphere contraction. Because CEF is a β-lactam antibiotic, it is particularly affected by diffusion barriers within biofilms, resulting in limited penetration and entrapment in outer layers. For CIP (Figure 4B), substantial microsphere shrinkage and left-shifted distribution appeared at 24 h, followed by a partial recovery at 36 h. Such a rebound could signify resistant subpopulation development, consistent with CIP’s mechanism (DNA gyrase inhibition) and superior biofilm penetration [34,35,36]. At 50 μg/mL, microsphere shrinkage reached a plateau, suggesting almost full inhibition by CIP. Figure 4C illustrates that AMP caused only gradual diameter changes regardless of dosage. Even the highest AMP dose (200 μg/mL) failed to fully suppress biofilm activity, reflecting the high tolerance of mature biofilms. The weaker effect of ampicillin (AMP) compared with ceftriaxone (CEF) is likely attributable to its preferential activity against actively dividing cells through inhibition of cell wall synthesis, whereas bacteria within biofilms often exhibit slow growth or metabolic dormancy, rendering them intrinsically less susceptible to β-lactam antibiotics [37]. Overall, Figure 4 highlights 24–36 h as the key interval for distinguishing biofilm sensitivity versus tolerance based on diameter shifts. Each antibiotic caused dose-dependent contraction between 24 and 36 h, with distinct patterns driven by differences in penetration and pharmacological mechanisms. Even under high antibiotic exposure, the preserved microsphere structure indicates that the mature biofilm matrix restricts antibiotic access and function, thereby safeguarding resident bacteria. These results are consistent with earlier studies describing the extreme antibiotic tolerance of bacterial biofilms. Additionally, this system delivers a geometric, image-based metric that complements conventional biological evaluations.

Collectively, the data show that biofilms formed on GelMA microspheres exhibit markedly elevated antibiotic tolerance compared with the reported planktonic MIC ranges for *E. coli* MG1655 in the literature, highlighting the well-established disparity between planktonic susceptibility and biofilm-associated tolerance (Table 1) [38,39,40]. These measurements serve as baseline benchmarks for biofilm-oriented antibiotic susceptibility assays.

### 2.5. Estimating the Biofilm MIC by Monitoring Diameter Variations in GelMA Microspheres

**Figure 5:**
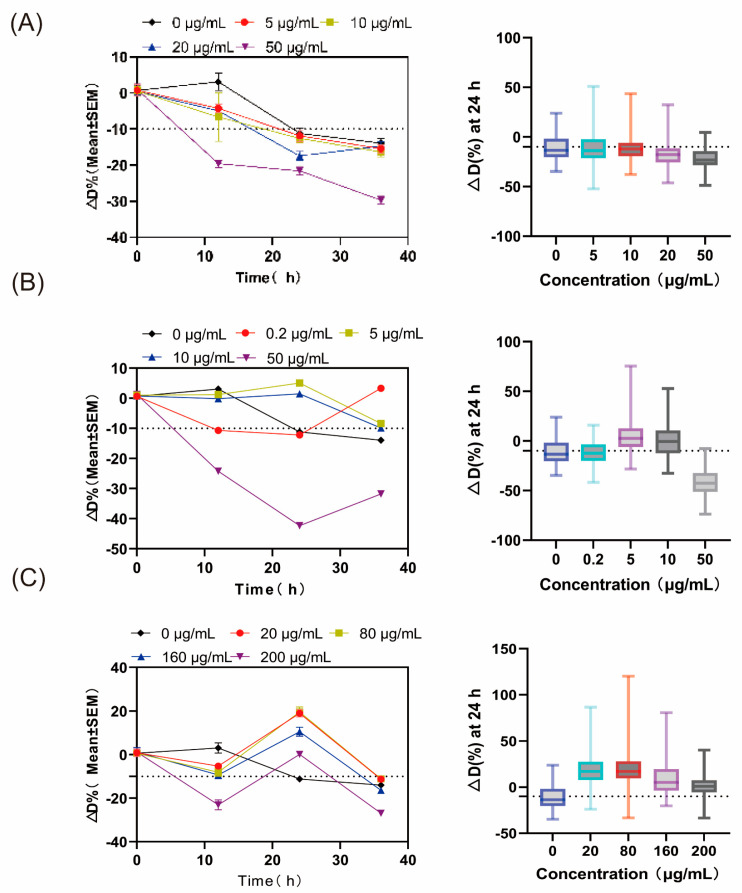
Biofilm MBIC estimation based on particle size variations in GelMA microspheres under antibiotic exposure. *E. coli* MG1655-laden microspheres were subjected to exposure to (**A**) ceftriaxone, (**B**) ciprofloxacin, and (**C**) ampicillin. The left panel shows the relationship between ΔD% (mean ± SEM) and time (hours) on the y-axis. The right panel shows the distribution of ΔD% at 24 h for each specified dose (box plot with whiskers). The gray dashed line (ΔD = −10%) indicates the operational threshold for defining biofilm MBIC in this study. Around 100 microspheres were analyzed in parallel for each condition; only those microspheres meeting pre-specified sphericity criteria were considered for analysis.

In addition, we employed diameter-change trajectories as an operational readout to estimate the minimum biofilm inhibitory concentration. Calculation of ΔD% utilized the mean baseline (0 h) diameter corresponding to each antibiotic dose as $d_{0}$. Furthermore, a threshold of ΔD% = −10% was determined using the antibiotic-free, bacteria-associated microsphere group. A given concentration was classified as the MBIC if its ΔD% fell to or remained below the threshold between 24 and 36 h and exhibited a markedly left-shifted trajectory at 24 h relative to lower-dose groups. Figure 5 illustrates that all tested antibiotics induced dose-dependent downward displacement in ΔD% trajectories. As depicted in Figure 5A, ceftriaxone caused dose-dependent reductions in ΔD% at 24 h, with concentrations of 20–50 μg/mL exceeding the −10% threshold. For ciprofloxacin, the ΔD% dropped sharply at 24 h—likely reflecting its mechanism of DNA gyrase inhibition, peak-dependent bactericidal activity, and rapid biofilm penetration enabled by low molecular mass and hydrophobicity—yet recovery at 36 h suggests the development of resistant subpopulations. Additionally, the 50 μg/mL dose fell substantially below the −10% threshold (Figure 5B). For ampicillin, microsphere shrinkage varied, and ΔD% never crossed the −10% threshold, possibly reflecting slow penetration and stress-mediated EPS upregulation. However, a dose-responsive decline was still evident (Figure 5C). Using this standard, the calculated minimum biofilm inhibitory concentration values were CEF 20–50 μg/mL, CIP 10–50 μg/mL, and AMP ≥ 200 μg/mL. All values exceeded the corresponding planktonic MICs, aligning with the elevated tolerance characteristic of biofilm-embedded bacteria. These findings indicate that diameter-response metrics offer a scalable, intuitive pharmacodynamic indicator for comparing antibiotics and optimizing dosages.

**Figure 6:**
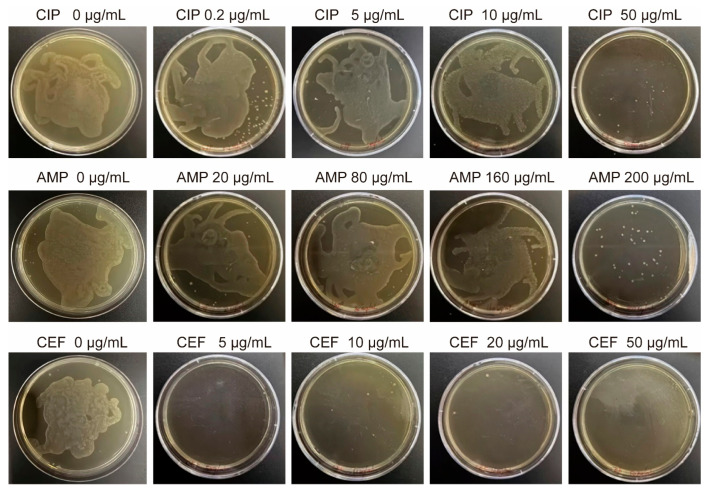
Bright-field images of Petri dishes after plating and 24 h of incubation, following 24 h exposure of microspheres to varying concentrations of three antibiotics. The culture medium remained clear in all wells, indicating higher MIC for biofilm-embedded bacteria compared to planktonic cultures.

Because the MIC of biofilm-embedded bacteria is substantially higher than that of planktonic cultures, the culture medium in all wells remained visibly clear. We collected microcarriers incubated with bacteria under different antibiotic conditions at the 24-h time point (when the particle size variation was most significant). The microspheres were then disrupted, centrifuged, and plated on agar plates for cultivation to observe bacterial growth and viability under biofilm conditions (Figure 6). During the initial 8 h, plates containing antibiotics showed almost no colony growth, while plates inoculated with disrupted microspheres from the no-antibiotic group exhibited visible colonies. Following 24 h incubation, colonies were evident in CIP-treated groups at 0.2–50 μg/mL, while the 50 μg/mL group exhibited almost no growth, aligning well with the microsphere-size analysis. Likewise, AMP treatment resulted in nearly complete absence of colonies at the maximal tested concentration of 200 μg/mL. Another notable observation was that CEF-treated microspheres failed to yield any colonies after 24 h incubation. We hypothesize that CEF exposure induced bacteria into the viable but non-culturable (VBNC) state [41]. Under such conditions, the antibiotic may not have eradicated the bacteria but rather triggered a survival mode in which culturability is lost. The plate-culture outcomes closely corresponded with the findings from microsphere-based measurements.

This work introduces a label-free quantitative approach to evaluate biofilm-associated antibiotic susceptibility by tracking the dynamic size variations in GelMA microspheres, enabling direct correlation between *E. coli* activity in a 3D hydrogel environment and the minimum biofilm inhibitory concentration assessment. Relative to conventional biofilm evaluation systems, the proposed platform provides markedly enhanced measurement dimensionality and analytical resolution. Widely applied methods for biofilm susceptibility testing, including microplate-based crystal violet assays, metabolic dyes (XTT, resazurin), MBEC platforms, flow-cell systems, and fluorescence imaging, all depend on exogenous dyes, destructive handling, or single-timepoint readouts. Such assays cannot effectively separate growth inhibition from biomass loss, nor do they allow real-time monitoring of biofilm responses to antibiotics or capture the inherent heterogeneity, diffusion limitations, and tolerant subpopulations within 3D biofilms [42]. Recent studies have suggested that although hydrogel-based models begin to approximate in vivo viscosity and diffusion constraints, their ability to resolve the minimum biofilm inhibitory concentration assessment remains limited, and they typically lack real-time, non-perturbative kinetic readouts. By comparison, the GelMA-based 3D microsphere platform naturally offers adjustable porosity, defined mechanical properties, and an ECM-mimicking niche, allowing bacteria to establish dispersed and heterogeneous microcolonies that more faithfully reproduce key clinical biofilm traits. Additionally, bright-field imaging allowed real-time observation of microsphere size dynamics without reliance on staining or destructive sample preparation. The method eliminates potential biases from dye toxicity and chemical perturbation while providing high-throughput, single-particle diameter distributions via automated imaging, allowing sensitive detection of tolerant subpopulations and their time-dependent dynamics. The microfluidics-based setup further enables simultaneous evaluation across antibiotic concentration gradients, granting high scalability and full automation in biofilm susceptibility testing.

Finally, this study completed the preliminary validation of the platform using *Escherichia coli*—a typical rod-shaped Gram-negative bacterium—as the model strain. However, bacteria exhibit high diversity in morphology and structure. Gram-positive cocci represented by the genus *Staphylococcus*, characterized by a thick cell surface peptidoglycan layer and clustered growth, show significant differences from rod-shaped bacteria in both packing density and mechanical interaction with the gel matrix. These differences will directly affect microsphere deformation kinetics and bacterial antibiotic response characteristics.

Therefore, future research will expand the strain scope of this platform by incorporating bacteria of various morphologies and Gram properties, such as cocci and spirilla. We will systematically investigate the regulatory effects of bacterial morphology on detection parameters, further optimize platform design, comprehensively evaluate its versatility and robustness, and provide technical support for antibiotic susceptibility testing of multiple types of clinical bacterial infections.

## 3. Conclusions

In conclusion, we established a high-throughput platform capable of assessing antibiotic susceptibility under biofilm-associated conditions. The approach is based on microspheres that support the formation of dense bacterial biofilms, recapitulating essential in vivo features such as structural architecture and antibiotic sensitivity profiles. Bacterial viability after antibiotic exposure was inferred by quantifying microsphere diameter alterations. Beyond improving detection throughput, the platform offers a robust tool for probing biofilm-associated resistance mechanisms and identifying potential antimicrobial candidates. Additionally, the technique holds promise for refining antibiotic dosage strategies, enhancing therapeutic efficacy, and supporting the development of personalized treatment approaches. We anticipate that the proposed method will work synergistically with conventional AST assays and rapid diagnostic modalities—such as droplet microfluidic systems designed to capture rare resistant variants—thus providing significant opportunities for continued engineering development and clinical validation.

## 4. Materials and Methods

### 4.1. Materials and Reagents

Polydimethylsiloxane (PDMS-FF-200) chips, fluorocarbon oil (HFE7500), charge-coupled device cameras, and multi-channel pressure pumps were purchased from FluidicLab (Shanghai, China). Ampicillin, ciprofloxacin, and ceftriaxone were obtained from Sigma-Aldrich (Merck, Germany). GelMA (EFL-GM-PR-001) and lithium phenyl-2,4,6-trimethylbenzoylphosphinate (LAP) were sourced from Engineering for Life (Suzhou, China).

### 4.2. Preparation of Gelatin Microspheres Using a Microfluidic Approach

This study employs microfluidic flow focusing technology to fabricate gelatin methacryloyl (GelMA) microspheres within polydimethylsiloxane (PDMS) devices. A 3% (*w*/*v*) GelMA mixed with 0.25% photoinitiator lithium phenyl-2,4,6-trimethylbenzoylphosphinate (LAP) solution was used as the aqueous phase. The aqueous and oil phases (fluorocarbon oil) were introduced into a microfluidic droplet generator using a pressure-driven pump, with applied pressures of 100 mbar and 150 mbar, respectively. The generated water-in-oil droplets were collected in Eppendorf tubes. The droplets were subsequently photo-crosslinked under 405 nm light for 1 min. After crosslinking, the droplets were washed twice with a demulsifier to break the emulsion, followed by thorough rinsing with phosphate-buffered saline (PBS) to remove residual oil. Finally, 3% (*w*/*v*) GelMA microspheres were obtained. The microspheres were stored in sterile phosphate-buffered saline (PBS) at 4 °C and used within 48 h for all experiments.

### 4.3. Bacterial Cultivation and Biofilm Development

*Escherichia coli* K-12 MG1655, preserved at −80 °C in 10% (*v*/*v*) dimethyl sulfoxide (DMSO), was streaked onto Luria–Bertani (LB) agar plates and incubated at 37 °C for 16–18 h. A single colony was then inoculated into LB broth and cultured at 37 °C with shaking at 200 rpm until reaching the logarithmic growth phase (OD600 ≈ 0.4–0.6). Cells were harvested by centrifugation at 5000× *g* for 5 min, washed twice with sterile phosphate-buffered saline (PBS), and resuspended in PBS to a final concentration of 1 × 10^8^ CFU/mL. Subsequently, 300 μL of 3% (*w*/*v*) GelMA microspheres was added to the bacterial suspension and gently mixed. After bacterial seeding, the GelMA microspheres were incubated at 37 °C under static conditions for 16–24 h to allow biofilm formation [43]. This incubation period corresponds to the early-to-intermediate stages of *E. coli* biofilm development, during which bacterial adhesion and extracellular matrix production are established.

### 4.4. Antibiotic Concentration Settings

To evaluate biofilm-associated antibiotic responses, *E. coli* MG1655 biofilms formed on GelMA microspheres were exposed to antibiotics at predefined concentration ranges. Based on preliminary dose–response observations, ceftriaxone (CEF) was tested at concentrations ranging from 0 to 50 μg/mL, ciprofloxacin (CIP) from 0 to 50 μg/mL, and ampicillin (AMP) from 0 to 200 μg/mL.

### 4.5. Disruption and Colony-Forming Unit (CFU) Assay

After 24 h of antibiotic treatment, 1.5 mL of microspheres was transferred to centrifuge tubes and spun at 2000× *g* for 5 min. The supernatant was discarded, and the microspheres were mechanically disrupted by vigorous pipetting using sterile pipette tips until they were fully broken or substantially reduced in size. The resulting suspensions were serially diluted tenfold in sterile PBS. Aliquots (100 μL) of appropriate dilutions were then spread onto LB agar plates. After absorption, the plates were inverted and incubated at 37 °C for 16–24 h. Colonies were counted manually, and CFU values were calculated by multiplying the colony number by the corresponding dilution factor.

### 4.6. Microscopy Analysis

The adhesion of *E. coli* MG1655 to microsphere bead surfaces was visualized using scanning electron microscopy (SEM). The microspheres were fixed in 3% glutaraldehyde for 90 min, followed by sequential ethanol dehydration from 50% to 100%, with each concentration applied for 15 min. Critical point drying was subsequently carried out (Leica EM CPD300, Wetzlar, Germany). The samples were affixed to stubs with conductive carbon tape, sputter-coated with gold for 100 s, and imaged on a Hitachi SU8600 (Tokyo, Japan) scanning electron microscope. It should be noted that fixation and dehydration were applied only for SEM observation, while microsphere size and integrity were evaluated under hydrated conditions.

### 4.7. Culture of Microspheres and Cells Under Varying Antibiotic Concentrations

In a 24-well plate, each well received 1 mL of *E. coli* MG1655 suspension at 1 × 10^8^ CFU/mL, together with 10 μL of microspheres. Antibiotic gradients were prepared according to the planktonic MICs, and each concentration was tested in triplicate [44]. Cultures were incubated, and hydrogel beads were imaged at 0, 24, and 36 h using a CytoSMART Lux2 system (Eindhoven, The Netherlands) equipped with a 10× objective.

### 4.8. Statistical Analyses and Evaluation of Antibiotic Resistance

The diameters of microspheres were quantified with ImageJ 1.53k software. To quantify the dispersion of microsphere size measurements around the mean, the coefficient of variation (*CV*) was calculated as follows:

(1) $$CV(\%)=\frac{\sigma }{\mu }\times 100\%$$

where σ denotes the standard deviation (μm) and μ corresponds to the mean diameter (μm).

To analyze trends in microsphere diameter changes, the percentage diameter change (Δ*D*) was determined using the following equation:

(2) $$∆D\left(\%\right)=\left[{\left(\frac{d_{t}}{d_{0}}\right)}^{3}-1\right]\times 100\%$$

where $d_{t}$ denotes the average microsphere diameter (µm) at time t, while $d_{0}$ represents the mean initial diameter at t = 0 (µm). The minimum antibiotic concentration causing a marked decrease in hydrogel bead size (ΔD < −10%) was considered the antibiotic tolerance threshold for the biofilm state. This threshold was empirically defined to represent a biologically meaningful structural response that exceeded experimental variability and background fluctuations. Similar operational thresholds have been widely used in biofilm and biomaterial-based assays to define treatment-induced structural or viability changes when absolute killing is not directly measured.

## Figures and Tables

**Table 1:** Estimated minimum biofilm inhibitory concentration (MBIC, μg/mL) of *E. coli* MG1655 in the GelMA microsphere biofilm model.

Bacterial Strains	Antibiotics (μg/mL)		
	Ceftriaxone	Ciprofloxacin	Ampicillin
*Escherichia coli* MG1655-MBIC	20–50	10–50	≥200
*Escherichia coli* MG1655-MIC	0.06	0.008	1.5–2.5

≥ indicates the minimum dose at which stable inhibition is observed.

## Data Availability

The original contributions presented in this study are included in the article material. Further inquiries can be directed to the corresponding authors.

## Abbreviations

The following abbreviations are used in this manuscript:

MIC	minimum inhibitory concentration
CEF	ceftriaxone
CIP	ciprofloxacin
AMP	ampicillin
